# Psychometric Analysis of the eHealth Literacy Scale in Portuguese Older Adults (eHEALS-PT24): Instrument Development and Validation

**DOI:** 10.2196/57730

**Published:** 2025-02-26

**Authors:** Sara Luz, Paulo Nogueira, Andreia Costa, Adriana Henriques

**Affiliations:** 1 Nursing Research, Innovation and Development Centre of Lisbon (CIDNUR) Nursing School of Lisbon (ESEL) Lisbon Portugal; 2 Enviromental Health Institute (ISAMB) Faculty of Medicine University of Lisbon Lisbon Portugal; 3 Public Health Research Centre, Comprehensive Health Research Center (CHRC) NOVA National School of Public Health NOVA University of Lisbon Lisbon Portugal

**Keywords:** eHealth, health literacy, older adults, psychometric properties, public health

## Abstract

**Background:**

In this era of digitalization, eHealth interventions are used to engage patients in health care and help them manage their health. Previous studies showed that this can be particularly interesting for chronic disease self-management and self-care in older adults. Despite older adults becoming increasingly active on the internet, they continue to struggle in using eHealth information due to inadequate eHealth literacy. Thus, assessing and monitoring eHealth literacy is critical to support eHealth interventions.

**Objective:**

This study aimed to describe the translation, adaptation, and validation process of the eHealth Literacy Scale (eHEALS) in Portuguese older adults.

**Methods:**

The cross-cultural adaption followed the steps of forward and blinded backward translations, evaluation of the translations by a committee of judges, pilot-testing, and full psychometric testing. We tested the psychometric properties of the eHEALS by carrying out two studies: general psychometric analysis (study 1) and confirmatory factor analysis (study 2). Study 1 included 80 older adults conveniently selected from a Health Family Unit. Data were collected by in-person questionnaires between May and July 2022. Study 2 included 301 older adults randomly selected from two distinct Health Family Units. Data were collected by in-person questionnaires between May and July 2023.

**Results:**

We tested stability, reliability, construct validity (exploratory and confirmatory factor analyses and known groups), and model fit. Study 1 had 58.8% (47/80) male and 41.3% (33/80) female respondents (mean age 71.20, SD 5.26 years). Study 2 had 56.5% (170/301) male and 43.5% (131/301) female respondents (mean age 71.77, SD 5.15 years). Moderate and strong correlations were identified in the scale items (study 1: 0.42≤*r*≤0.91 and study 2: 0.81≤*r*≤0.96; *P*<.001). The scale showed good internal consistency for study 1 (α=.92) and study 2 (α=.98), with high correlations between items. The exploratory factor analysis yielded a single-factor structure, explaining 58.3% of the variance in study 1 and 86.4% in study 2. In the confirmatory analysis (study 2), the model fit was mixed (*χ*²_20_=265, *P*<.001; comparative fit index=0.94; Tucker-Lewis Index=0.91; root mean square error of approximation=0.20). Thus, we compared 1-, 2-, and 3-factor structures, deciding on the unidimensional one. In study 1, the eHEALS-PT24 (Portuguese version of the eHealth Literacy Scale for older adults) mean score was 27.25 (SD 5.61), with 43.8% (35/80) and 11.3% (9/80) of participants showing low and high eHealth literacy levels, respectively. In study 2, the eHEALS-PT24 mean score was 23.31 (SD 9.53), with 38.2% (115/301) and 23.6% (71/301) of participants showing low and high eHealth literacy levels, respectively. The known-groups analysis showed statistically significant differences between eHealth literacy and demographic variables (*P*<.001).

**Conclusions:**

The findings suggest that the eHEALS-PT24 is a reliable and valid tool to assess eHealth literacy in Portuguese older adults. Therefore, this instrument can be integrated to support the implementation process of eHealth interventions.

## Introduction

### Background

Currently, the internet is the main source of health information. According to the survey Flash Eurobarometer 404 [1], 8 (80%) in 10 Europeans used the internet for private matters, with the majority (59%) using it for health information searching purposes. The increasing number of internet users has been influencing health systems globally, leading to investments in the provision of eHealth services. In recent years, there have been great efforts to use eHealth interventions to engage patients in health care and help them manage their health, particularly among older adults with chronic disease self-management and self-care [2-4]. Nevertheless, despite the broad access to the internet and other electronic sources, there continues to be a lack of skills to access valid and reliable health information, as well as to understand, evaluate, and apply it adequately for decision-making purposes [5-7]. Considering this condition, eHealth literacy has emerged as a new concept in health care and a challenge in public health [8].

According to Norman and Skinner [9], eHealth literacy is “the ability to seek, find, understand, and evaluate information from electronic sources and apply it to knowledge gained to address or solve a health problem.” Based on this, individuals should be able to perform basic or advanced information retrieval, distinguish documents from reliable sources (eg, authoritative ones or scientific evidence-based), and understand eHealth terminology. Furthermore, this set of skills requires the ability to use information and communication technology (ICT), think critically about their nature, and efficiently navigate different electronic resources to obtain information for health-related decision-making.

Considering that eHealth literacy is not a static set of skills, instead changing over time, Norman and Skinner [9] consider the importance of assessing and monitoring eHealth literacy as critical to supporting eHealth interventions. In recent years, indeed, older adults have become increasingly active on the internet and interested in digital health care services to manage their health; however, limited attention has been given to measuring eHealth literacy in this population. Therefore, we sought to address this gap by translating, culturally adapting, and validating a scale for Portuguese older adults that measure eHealth literacy levels (Multimedia Appendix 1).

### The eHealth Literacy Scale

The eHealth Literacy Scale (eHEALS), developed by Norman and Skinner [10], was the most frequently used instrument to measure eHealth literacy worldwide, covering 18 languages, 26 countries, and diverse populations and contexts (ie, adolescents, adults, elderly, patients, healthy people, caregivers, health school professionals, community, and clinical practice) [11]. Since its development, the eHEALS has been widely translated, adapted, and validated in several countries, such as Brazil [12], China [13-16], Ethiopia [17], Germany [18,19], Greece [20], Hungary [21], Indonesia [22], Iran [23,24], Italy [25,26], the Netherlands [27]; Norway [28,29], Poland [30,31], Portugal [32], Serbia [33], South Korea [34-36], Spain [37], and Sweden [38], as well as in countries with English as the main language [39-47].

The scale’s development study [10] assessed the eHealth literacy of Canadian adolescents (n=664) aged between 13 and 21 years from 14 secondary schools. Considering the psychometrics test results, the instrument showed a good internal consistency (α=.88), with moderate to strong correlations between items (0.51≤*r*≤0.76) and a moderate test-retest reliability (0.40≤*r*≤0.68). In addition, a unidimensional structure was found in exploratory and confirmatory factor analyses, explaining 56% of the total variance.

In Portugal, and similarly to the original study, the eHEALS was translated, adapted, and validated in 2014 in a sample of adolescents (n=1215) attending secondary education. The results showed a good internal consistency (α=.84) and a 2-factor structure in the exploratory factor analysis (EFA; extraction of 2 factors with α=.81 and α=.73), explaining 61% of the total variance. From a subgroup analysis, the authors found statistically significant differences regarding the level of education. For the variables sex and age, no statistically significant differences were found. No further translation, adaptation, and validation studies of eHEALS considering other target populations or contexts in Portugal have been carried out since then [32].

Studies with samples including older adults [24,36,39,43,44,48] showed good internal consistency, with Cronbach α ranging between 0.87 and 0.99. Regarding factor analysis, 3 studies [24,36,39] obtained 1D structures, and 2 other studies obtained 3D ones [43,44].

The questionnaire itself consists of 8 items assessing the person’s perception of knowledge, comfort, and ability to find, evaluate, and apply health ICT. Each of the items is scored at 5 points on a Likert scale ranging between “1” (totally disagree) and “5” (totally agree), with a total score from 8 to 40 points. The higher the score, the higher the eHealth literacy levels. Items 1 and 2 are related to awareness, items 3 and 4 are related to demand, items 6 and 7 are related to the evaluation of health resources, and items 5 and 8 are related to the use of health information. The instrument also contains 2 additional items, not adding to the final score, which assesses the participant’s perception of using the internet to access health information and make health decisions in terms of its usefulness and importance. The theoretical basis of the eHEALS was the social cognitive theory of Albert Bandura and the Lily Model, which explains multiple components of the constructs based on 6 components of literacies—traditional (literacy and numeracy skills), health, information, scientific, media, and computer [10].

This paper presents and discusses the results of full psychometric testing of the eHEALS-PT24 (Portuguese version of the eHealth Literacy Scale for older adults), which aimed to:

- Translate, culturally adapt, and validate the eHEALS for assessing eHealth literacy in Portuguese older adults.
- Test full psychometric characteristics of the eHEALS to be used in Portuguese older adults.
- Explore associations between eHealth literacy and sociodemographic variables.

## Methods

### Design

**Figure 1:**
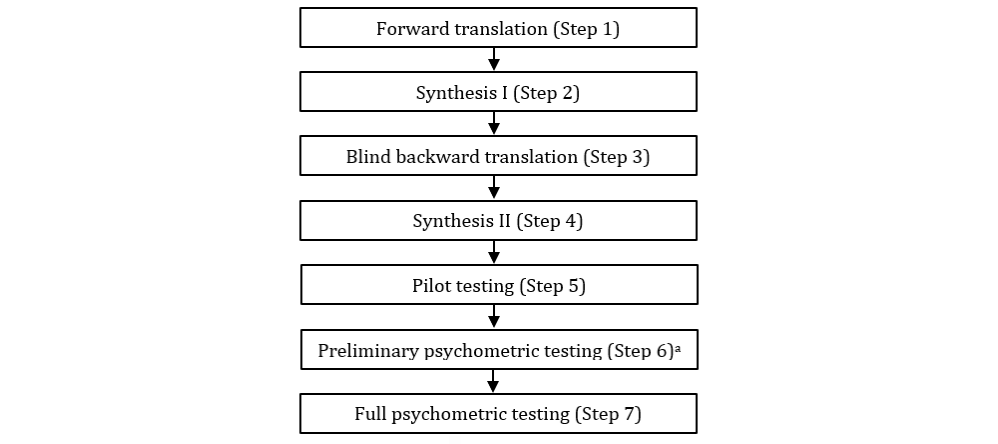
Steps of the protocol followed for translating, cultural adapting, and validating the eHealth Literacy Scale (eHEALS). ^a: N/A: not applicable.^.

We followed a methodology for translating, adapting, and validating instruments proposed by Sousa and Rojjanasrirat [49]—a 7-step guideline based on the review of existing recommendations in the scientific literature on the process of translation, cultural adaptation, and validation of instruments for use in cross-cultural health care (Figure 1).

The original version of the questionnaire was independently translated to Portuguese by 2 translators with high proficiency in English: a translator with knowledge of the terminology used in health care and another with knowledge of the cultural and linguistic characteristics of the target language (forward translation; step 1). After this, a third translator compared the two translated versions of the instrument and the original one to detect discrepancies. In addition, a first meeting with a committee of experts with different backgrounds and expertise was held to evaluate discrepancies, composed of the 2 bilingual experts from the first step, the third bilingual translator, academia members, and the research team (step 2). These 2 steps generated the preliminary translated version of the instrument for the Portuguese language.

Independent, blinded backward translation to English was carried out by 2 translators whose first language was English as spoken in the United Kingdom: one translator with knowledge of the terminology used in health care and another with knowledge of the cultural and linguistic characteristics of the target language (step 3). This step generated 2 retroverted versions of the original version of the instrument, which were then compared with the original one (in English) to detect discrepancies by the research team. In addition, a second meeting with a committee of experts with different backgrounds and expertise was held to detect discrepancies, composed of all translators involved in the previous three steps, academia members, and the research team (step 4). These 2 steps generated the prefinal version of the translated instrument, which the original authors validated.

The prefinal version of the instrument was then tested in a pilot study in the target language with a monolingual sample (n=15) to evaluate the instructions, items, and response format clarity (ie, to establish whether the instrument could be satisfactorily understood and completed by people from the target population; step 5). As part of this step, a committee of experts (n=8) was also held to further examine the instrument for clarity of the instructions, items, and response format (content equivalence assessment). The fifth step generated some adjustments in the prefinal version of the instrument.

According to the protocol followed, step 6 (preliminary psychometric testing of the prefinal version of the translated instrument with a bilingual sample) is rarely used, except when a bilingual population is accessible, which justified the option of not carrying it out in this validation process.

Full psychometric testing (step 7) involved reviewing and refining the items of the final version of the eHEALS for Portuguese older adults, as well as establishing stability, internal consistency, validity, and model fit. This step encompassed 2 substudies: general psychometric characteristics of the instrument (n=80, study 1) and confirmatory factor analysis (CFA; n=301, study 2). The sample sizes were defined according to the protocol, namely, at least 10 participants per item of the instrument for general psychometric analysis and 300 to 500 participants for CFA.

### Participants and Recruitment

Study 1 included 80 older adults from a Health Family Unit (Primary Care) located in Faro. The sampling procedures were based on a convenience sample. Data collection was conducted by in-person questionnaires between May and July 2022. During that time, patients aged 65 years or older who went to the Health Family Unit (Primary Care) for an appointment were invited to participate in the survey.

Study 2 included 301 older adults from 2 distinct Health Family Units (Primary Care), located in Faro and Lisbon. Participants were randomly selected from the total number of patients of the 2 considered Health Family Units and invited by phone to visit the unit and participate in the survey. Data collection was conducted by in-person questionnaires between May and July 2023.

Inclusion criteria were age (aged 65 years or older) and sex (female and male). Difficulty communicating or using the Portuguese language was an exclusion criterion. The questionnaire removal was performed when the questionnaires were not entirely fulfilled.

### Data Analysis

The data analysis was conducted in several stages to ensure a comprehensive understanding of the psychometric properties of the eHEALS-PT24. The following steps outline our analytical approach.

#### Descriptive Statistics

Initially, we performed descriptive statistical analyses to understand the basic features of the data. This included calculating mean, SD, frequencies, and percentages for all the variables, providing a snapshot of the sample characteristics.

#### Scale and Items Stability

To assess the scale and item stability, we calculated Pearson correlations. We considered the following Pearson correlation ranges: very weak (0.00-0.19), weak (0.20-0.39), moderate (0.40-0.59), strong (0.60 and 0.79), and very strong (0.80-0.99). Also, statistical significance was set at *P*<.001 [50].

#### Reliability Analysis

To assess the internal consistency, we calculated Cronbach α coefficients. A Cronbach α value of 0.70 or higher was considered indicative of acceptable reliability. The item-total correlation between the items and the total score was considered high, with values above 0.40 [50].

#### Construct Validity: Factor Analysis

We conducted an EFA to explore the underlying factor structure of the scale. The Kaiser-Meyer-Olkin (KMO) measure and Bartlett test of sphericity were used to assess the suitability of the data for factor analysis. Factors were extracted using principal component analysis, and a varimax rotation was applied to aid interpretability.

Following EFA, a CFA was performed using structural equation modeling to verify the factor structure obtained from EFA. Model fit was evaluated using different indices, including the comparative fit index (CFI), Tucker-Lewis Index (TLI), root mean square error of approximation (RMSEA), and chi-square (*df*) ratio. RMSEA values of 0.00 to 0.05 indicate a closer or good fit, 0.05 to 0.08 a fair fit, 0.08 to 0.10 a mediocre fit, and over 0.10 a poor fit. Other fit indexes that we used were the CFI and TLI, both of which should be close to 0.95 [51].

#### Construct Validity: Known-Groups Validity

The known-groups validity measures an instrument’s ability to distinguish among distinct groups (ie, discriminating groups known to differ on the variable of interest) [50]. To evaluate the known-groups validity, we compared eHEALS-PT24 scores across different sociodemographic groups (ie, age, sex, residence area, and education level) using the independent Student *t* test (2-tailed), ANOVA, or Kruskal-Wallis test, as appropriate.

#### Statistical Software

All analyses were performed using IBM SPSS and Jamovi (The Jamovi Project). By using this multistep analytical process, we aimed to rigorously evaluate the psychometric properties of the eHEALS-PT24, ensuring its reliability and validity in assessing eHealth literacy among Portuguese older adults.

### Ethical Considerations

The ethics review assessments were submitted and approved by Portugal’s National Data Protection Authority and Regional Health Administrations (Algarve, Lisbon, and Tagus Valley; (approval 4825/2018). Informed consent was applied to participants, according to the model recommended by the Ethics Committee of the Algarve Regional Health Administration, guaranteeing voluntary participation, the possibility of withdrawing at any time, data anonymity, privacy, confidentiality, and no harm to participants. In addition, an authorization request was made to the original authors of the eHEALS [10], which was accepted.

## Results

### Pilot Study

Before the full psychometric testing, we carried out a pilot study, which we briefly summarized to support the results presented in this subsection. The sampling procedure for piloting was based on snowball sampling, composed of older people aged 65 years or older (n=15). Data collection was conducted using a web-based questionnaire between July and August 2020. The interrater agreement among the sample was above 80% for all items. As part of this step, a committee of experts (n=8) was also held to further examine the instrument for clarity of the instructions, items, and response format; also, to assess the content equivalence, content validity index at the item level (I-CVI=1) and at the scale level (S-CVI=1), as well as the Fleiss κ coefficient of agreement (κ=0.24; *P*<.001), were calculated.

### Participant’s Characteristics

#### Study 1

Out of the 80 participants, the sample comprised 47 male (58.8%) and 33 female (41.3%) participants, with a mean age of 71 (SD 5.26) years, ranging from 65 to 88 years. From the total sample (n=80), most of the participants were married or had civil partners (n=63, 78.8%), living with their spouse (n=65, 81.3%) in urban areas (n=70, 87.5%). In addition, most of the respondents were retired (n=69, 86.3%). Regarding formal education, this was analyzed according to the International Standard Classification of Education (ISCED), showing that the most general education level was upper secondary education—ISCED 3 (n=20, 25%), followed by primary education—ISCED 1 (n=17, 21.3%), and bachelor or equivalent degree—ISCED 6 (n=17, 21.3%). Concerning the current health status, most participants reported a diagnosed chronic condition (n=58, 72.5%), mainly high blood pressure (n=29, 36.3%), diabetes (n=25, 31.2%), and dyslipidemia (n=8, 10%). Regarding prescribed medication, 85% (n=68) of the sample answered that they usually take medicines, with 3 being the average number of medicines per person and 9 being the maximum number. Table 1 presents the sociodemographic data.

**Table 1:** Sociodemographic data.

Characteristics			Study 1 (n=80)		Study 2 (n=301)
Average age (years), mean (SD)			71.20 (5.26)		71.77 (5.15)
**Sex, n (%)**					
	Female	33 (41.3)		131 (43.5)	
	Male	47 (58.8)		170 (56.5)	
**Marital status, n (%)**					
	Single	1 (1.3)		11 (3.7)	
	Married or civil partner	63 (78.8)		215 (71.4)	
	Divorced	9 (11.3)		27 (9)	
	Separated	0 (0)		2 (0.7)	
	Widowed or surviving civil partner	7 (8.8)		46 (15.3)	
**Household status, n (%)**					
	Lives alone	9 (11.3)		52 (17.3)	
	Lives with spouse	65 (81.3)		203 (67.4)	
	Lives with spouse and other relatives	3 (3.8)		25 (8.3)	
	Lives with other relatives	3 (3.8)		21 (7)	
**Residence area, n (%)**					
	Urban area	70 (87.5)		267 (88.7)	
	Rural area	10 (12.5)		34 (11.3)	
**Formal education, n (%)**					
	ISCED^a^ 1 Primary education	17 (21.3)		116 (38.5)	
	ISCED 2 Lower secondary education	5 (6.25)		22 (7.3)	
	ISCED 3 Upper secondary education	20 (25)		51 (17)	
	ISCED 4 Post-secondary but not tertiary education	8 (10)		22 (7.3)	
	ISCED 5 Short-cycle tertiary education	8 (10)		27 (9)	
	ISCED 6 Bachelor or equivalent level	17 (21.3)		50 (16.6)	
	ISCED 7 Master or equivalent level	2 (12.5)		6 (2)	
	ISCED 8 Doctoral or equivalent level	3 (3.8)		7 (2.3)	
**Employment status, n (%)**					
	Employed	6 (7.5)		15 (5)	
	Self-employed	3 (3.8)		9 (3)	
	Retired	69 (86.3)		271 (90)	
	Unemployed	0 (0)		2 (0.7)	
	Fulfilling domestic tasks	1 (1.3)		3 (1)	
	Other	1 (1.3)		1 (0.3)	
**Diagnosis of a chronic condition, n (%)**					
	Yes	58 (72.5)		252 (83.7)	
	No	22 (27.5)		49 (16.3)	
**Chronic condition (top 3), n (%)**					
	Diabetes	25 (31.3)		179 (59.5)	
	Dyslipidemia	8 (10)		45 (15)	
	High blood pressure	29 (36.3)		116 (38.5)	
**Usual prescribed medication, n (%)**					
	Yes	68 (85)		279 (92.7)	
	No	12 (15)		22 (7.3)	
Number of medicines, mean			3.10		3.10

^a^ISCED: International Standard Classification of Education.

#### Study 2

The sample comprised 170 male (56.5%) and 131 female (43.5%) participants, with a mean age of 72 (SD 5.15) years, ranging from 65 to 88 years. From the total sample (n=301), most of the participants were married or had civil partners (n=215, 71.4%), living with their spouse (n=267, 88.7%) in urban areas (n=267, 88.7%). In addition, most of the respondents were retired (n=271, 90%). Regarding formal education, this was analyzed according to the ISCED, showing that the most general education level was primary education—ISCED 1 (n=116, 38.5%), followed by lower secondary education—ISCED 2 (n=51, 17%) and bachelor or equivalent degree—ISCED 6 (n=37, 12.3%). Concerning the current health status, most participants reported a diagnosed chronic condition (n=252, 83.7%), mainly diabetes (n=179, 59.5%), dyslipidemia (n=45, 15%), and high blood pressure (n=116, 38.5%). Regarding prescribed medication, 92.7% (n=279) of the sample answered that they usually take medicines, with 3 being the average number of medicines per person and 10 being the maximum number. In this study, 19 questionnaires were excluded since they were not entirely fulfilled. Table 1 presents the sociodemographic data of both studies.

### Stability

#### Study 1

From the stability analysis (Pearson correlation coefficient) of the eHEALS-PT24, correlations between all items of the scale were identified, ranging between moderate and strong (except for one very strong correlation), with statistical significance (*P*<.001). The lowest correlation value (*r*=0.42) was between items 1 and 8 (moderate correlation). In turn, the highest value (*r*=0.91) was a very strong correlation between items 2 and 3 (Table 2).

**Table 2:** Pearson correlations for study 1.

			Item 1		Item 2	Item 3		Item 4		Item 5		Item 6		Item 7		Item 8
**Item 1**																
	*r*	1		0.66^a^		0.59^a^	0.60^a^		0.59^a^		0.44^a^		0.51^a^		0.42^a^	
	*P* value	—^b^		<.001		<.001	<.001		<.001		<.001		<.001		<.001	
**Item 2**																
	*r*	0.66^a^		1		0.91^a^	0.71^a^		0.69^a^		0.61^a^		0.52^a^		0.46^a^	
	*P* value	<.001		—		<.001	<.001		<.001		<.001		<.001		<.001	
**Item 3**																
	*r*	0.59^a^		0.91^a^		1	0,63^a^		0.65^a^		0.54^a^		0.45^a^		0.47^a^	
	*P* value	<.001		<.001		—	<.001		<.001		<.001		<.001		<.001	
**Item 4**																
	*r*	0.60^a^		0.71^a^		0,63^a^	1		0.76^a^		0.60^a^		0.64^a^		0.42^a^	
	*P* value	<.001		<.001		<.001	—		<.001		<.001		<.001		<.001	
**Item 5**																
	*r*	0.59^a^		0.69^a^		0.65^a^	0.76^a^		1		0.55^a^		0.55^a^		0.45^a^	
	*P* value	<.001		<.001		<.001	<.001		—		<.001		<.001		<.001	
**Item 6**																
	*r*	0.44^a^		0.61^a^		0.54^a^	0.60^a^		0.55^a^		1		0.70^a^		0.46^a^	
	*P* value	<.001		<.001		<.001	<.001		<.001		—		<.001		<.001	
**Item 7**																
	*r*	0.51^a^		0.52^a^		0.45^a^	0.64^a^		0.55^a^		0.70^a^		1		0.47^a^	
	*P* value	<.001		<.001		<.001	<.001		<.001		<.001		—		<.001	
**Item 8**																
	*r*	0.42^a^		0.46^a^		0.47^a^	0.42^a^		0.45^a^		0.46^a^		0.47^a^		1	
	*P* value	<.001		<.001		<.001	<.001		<.001		<.001		<.001		—	

^a^The correlation is significant at a significance level of .01 (2-tailed).

^b^Not applicable.

#### Study 2

From the stability analysis (Pearson correlation coefficient) of the eHEALS-PT24, correlations between all items of the scale were identified, ranging between moderate and strong (except for one very strong correlation), with statistical significance (*P*<.001). The lowest correlation value (*r*=0.81) was between items 1 and 8 (strong correlation). In turn, the highest value (*r*=0.96) was a very strong correlation between items 2 and 3 (Table 3).

**Table 3:** Pearson correlations for study 2.

			Item 1		Item 2		Item 3		Item 4		Item 5		Item 6		Item 7		Item 8
**Item 1**																	
	*r*	1		0.91^a^		0.90^a^		0.87^a^		0.87^a^		0.83^a^		0.82^a^		0.81^a^	
	*P* value	—^b^		<.001		<.001		<.001		<.001		<.001		<.001		<.001	
**Item 2**																	
	*r*	0.91^a^		1		0.96^a^		0.90^a^		0.88^a^		0.86^a^		0.83^a^		0.83^a^	
	*P* value	<.001		—		<.001		<.001		<.001		<.001		<.001		<.001	
**Item 3**																	
	*r*	0.90^a^		0.96^a^		1		0.90^a^		0.90^a^		0.86^a^		0.83^a^		0.83^a^	
	*P* value	<.001		<.001		—		<.001		<.001		<.001		<.001		<.001	
**Item 4**																	
	*r*	0.87^a^		0.90^a^		0.90^a^		1		0.93^a^		0.87^a^		0.85^a^		0.83^a^	
	*P* value	<.001		<.001		<.001		—		<.001		<.001		<.001		<.001	
**Item 5**																	
	*r*	0.87^a^		0.88^a^		0.90^a^		0.93^a^		1		0.87^a^		0.85^a^		0.85^a^	
	*P* value	<.001		<.001		<.001		<.001		—		<.001		<.001		<.001	
**Item 6**																	
	*r*	0.83^a^		0.86^a^		0.86^a^		0.87^a^		0.87^a^		1		0.87^a^		0.83^a^	
	*P* value	<.001		<.001		<.001		<.001		<.001		—		<.001		<.001	
**Item 7**																	
	*r*	0.82^a^		0.83^a^		0.83^a^		0.85^a^		0.85^a^		0.87^a^		1		0.85^a^	
	*P* value	<.001		<.001		<.001		<.001		<.001		<.001		—		<.001	
**Item 8**																	
	*r*	0.81^a^		0.83^a^		0.83^a^		0.83^a^		0.85^a^		0.83^a^		0.85^a^		1	
	*P* value	<.001		<.001		<.001		<.001		<.001		<.001		<.001		—	

^a^The correlation is significant at a significance level of .01 (2-tailed).

^b^Not applicable.

### Reliability

#### Study 1

The analysis of the internal consistency of the eHEALS-PT24 showed an adequate Cronbach α coefficient (Cronbach α=0.92). The statistics after excluding 1 of the 8 items did not indicate an increase in reliability: the value of Cronbach α ranged from 0.90 to 0.92. The mean total value in the eHealth literacy for the sample (n=80) was 27.25 (SD 5.61). The average score for each item was 3, ranging between 3.09 (item 8) and 3.55 (item 1). Concerning item-total correlation, coefficients above 0.4 for all items showed that the items were consistent with each other and correlated with the final score (Table 4).

**Table 4:** eHEALS-PT24^a^ means, scale reliability after removing an item, and item-total correlation.

Study and items		Mean (SD)	Mean if item deleted	α if item deleted	Variance of the scale if item deleted	Item-total correlation^b^
**Study 1 (n=80)**						
	Item 1	3.55 (0.80)	23.70	0.907	25.35	0.68
	Item 2	3.53 (0.87)	23.73	0.894	23.52	0.83
	Item 3	3.51 (0.87)	23.74	0.900	24.09	0.77
	Item 4	3.49 (0.94)	23.76	0.898	23.35	0.79
	Item 5	3.40 (0.88)	23.85	0.900	24.05	0.77
	Item 6	3.34 (0.94)	23.91	0.906	24.11	0.70
	Item 7	3.35 (0.89)	23.90	0.907	24.60	0.69
	Item 8	3.09 (0.87)	24.16	0.918	25.78	0.55
	Sum score, mean (SD)	27.25 (5.61)	—^c^	—	—	—
**Study 2 (n=301)**						
	Item 1	2.99 (1.27)	20.33	0.978	69.58	0.91
	Item 2	2.95 (1.30)	20.37	0.977	68.89	0.94
	Item 3	2.97 (1.28)	20.34	0.977	69.08	0.94
	Item 4	2.99 (1.30)	20.32	0.977	68.99	0.93
	Item 5	2.96 (1.28)	20.35	0.977	69.31	0.94
	Item 6	2.88 (1.26)	20.43	0.978	70.10	0.91
	Item 7	2.87 (1.24)	20.44	0.979	70.74	0.89
	Item 8	2.70 (1.22)	20.61	0.980	71.27	0.88
	Sum score, mean (SD)	23.31 (9.53)	—	—	—	—

^a^eHEALS-PT24: Portuguese version of the eHealth Literacy Scale for older adults.

^b^All item-to-total correlations were significant as *P*<.001.

^c^Not applicable.

#### Study 2

The analysis of the internal consistency of the eHEALS-PT24 showed an adequate Cronbach α coefficient (Cronbach α=0.98). The statistics after excluding 1 of the 8 items did not indicate an increase in reliability, with Cronbach α coefficient values remaining stable at 0.98. The mean total value in the eHealth literacy for the sample (n=301) was 23.31 (SD 9.53). The average score for each item was 3, ranging between 2.7 (item 8) and 2.99 (items 1 and 4). Concerning item-total correlation, coefficients above 0.40 for all items showed that the items were consistent with each other and correlated with the final score (Table 4).

### Construct Validity: EFA

The Bartlett sphericity test corroborated the factorability of the correlation matrix for both studies (study 1: *χ*^2^_28_=446.87, *P*<.001; study 2: *χ*^2^_28_=3932.81, *P*<.001). In addition, the KMO test value demonstrated adequate sampling for both studies (study 1: KMO=0.87; study 2: KMO=0.94). Given the quality of Bartlett and KMO values, the criteria for factor analysis were gathered. By performing the EFA, we considered factors with eigenvalues above 1 for testing the structure of the instrument. In accordance with the original structure of the eHEALS [10], the Jamovi software extracted 1 factor for the structure of the eHEALS-PT24 for both studies (Study 1 and 2).

#### Study 1

In the EFA of study 1 (n=80), a single factor showed moderate to strong loadings (0.57 to 0.89; Table 5) but poor model fit indicators: RMSEA was high (0.20), TLI was below the threshold (0.79), and there was a significant chi-square test (*χ*^2^_20_=83.7, *P*<.001). The factor explained 58.3% of the total variance.

**Table 5:** Factor loadings after varimax rotation.

eHEALS-PT24^a^ items	Study 1 (n=80), factor 1	Study 2 (n=301), factor 1
Item 1	0.72	0.92
Item 2	0.89	0.95
Item 3	0.82	0.95
Item 4	0.84	0.95
Item 5	0.81	0.95
Item 6	0.72	0.92
Item 7	0.70	0.90
Item 8	0.57	0.89

^a^eHEALS-PT24: Portuguese version of the eHealth Literacy Scale for older adults.

#### Study 2

In the EFA of study 2 (n=301), a single factor showed moderate to strong loadings (0.89 to 0.95) but poor model fit indicators: RMSEA was high (0.20), TLI was marginally acceptable (0.91), and there was a significant chi-square test (*χ*^2^_20_=271, *P*<.001). The factor explained 86.4% of the total variance.

**Figure 2:**
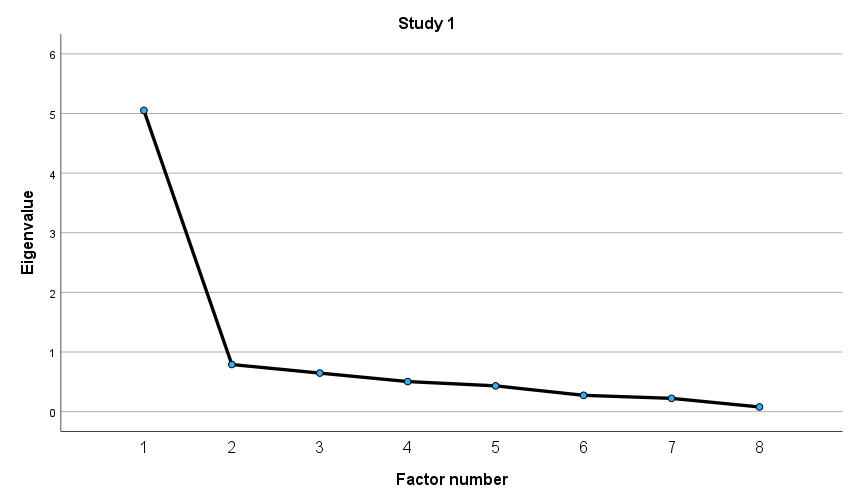
Screen plot for the eHEALS-PT24 (study 1). eHEALS-PT24: Portuguese version of the eHealth Literacy Scale for older adults.

**Figure 3:**
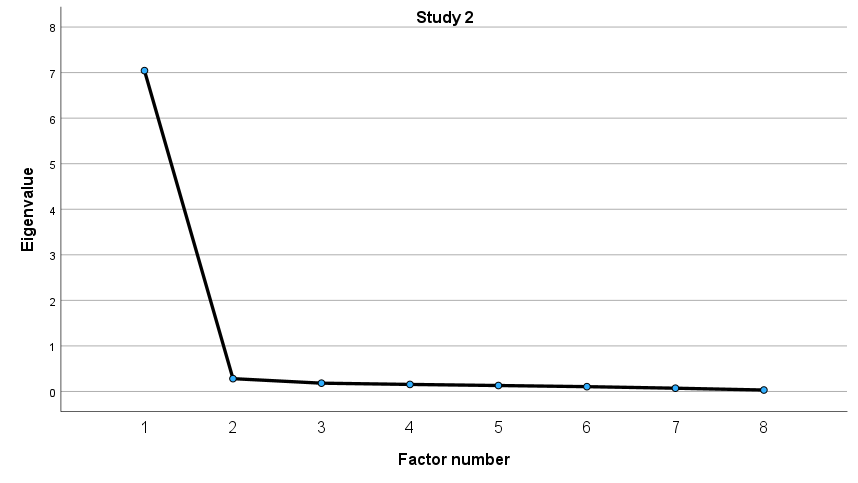
Screen plot for the eHEALS-PT24 (study 2). eHEALS-PT24: Portuguese version of the eHealth Literacy Scale for older adults.

The single-factor structure of eHEALS-PT24 for both studies (studies 1 and 2) was also empirically confirmed on screen plots (Figures 2 and 3).

### Construct Validity: CFA

Regarding CFA for study 2 (n=301), a single-factor model demonstrated high item loadings (standardized estimates 0.88 to 0.97; Table 6); however, the model fit was mixed: the chi-square test was significant (*χ*^2^_20_=265, *P*<.001), CFI was acceptable (0.94), while TLI (0.91) was marginally acceptable, and RMSEA (0.20) indicated a poor fit. Given the RMSEA values for one dimension, and due to the variability of factor structures in the literature, we compared the indexes for 1-, 2-, and 3-factor structures in study 2 (Table 7).

**Table 6:** Factor loadings after varimax rotation.

eHEALS-PT24^a^ items	Study 2 (n=301)	Study 2 (n=301)		Study 2 (n=301)		
	Factor 1	Factor 1	Factor 2	Factor 1	Factor 2	Factor 3
Item 1	0.93	0.93	—^b^	0.94	—	—
Item 2	0.96	0.97	—	0.98	—	—
Item 3	0.97	0.97	—	—	0.97	—
Item 4	0.94	0.94	—	—	0.94	—
Item 5	0.94	0.94	—	—	0.94	—
Item 6	0.91	—	0.93	—	—	0.93
Item 7	0.89	—	0.93	—	—	0.93
Item 8	0.88	—	0.90	—	—	0.90

^a^eHEALS-PT24: Portuguese version of the eHealth Literacy Scale for older adults.

^b^Not available.

**Table 7:** Global model fit indices.

Indices	1 Factor	2 Factors	3 Factors
RMSEA^a^ (90% CI)	0.20	0.17	0.18
CFI^b^	0.94	0.96	0.96
TLI^c^	0.91	0.94	0.93
Chi-square test, *P* value	<.001	<.001	<.001

^a^RMSEA: root mean square error of approximation.

^b^CFI: comparative fit index.

^c^TLI: Tucker-Lewis Index.

For the 2-factor model, we followed Tomás et al [32], dividing items 1-5 (factor 1) and items 6-8 (factor 2). High item loadings (standardized estimates 0.90 to 0.97) were obtained (Table 6); however, the model fit was mixed: CFI was acceptable (0.96), TLI (0.94) was marginally acceptable, RMSEA (0.17) indicated a poor fit, and the chi-square test was significant (*χ*^2^_19_=188, *P*<.001; Table 7).

For the 3-factor model, we followed Stellefson et al [43], dividing items 1-2 (factor 1), items 3-5 (factor 2), and items 6-8 (factor 3). High item loadings (standardized estimates 0.90 to 0.98) were obtained (Table 6); however, the model fit was mixed: CFI was acceptable (0.96), TLI (0.93) was marginally acceptable, RMSEA (0.18) indicated a poor fit, and the chi-square test was significant (*χ*^2^_17_=179, *P*<.001; Table 7).

### Interpretation of the eHEALS-PT24 Scores

We used the mean total and SD values to determine high or low eHealth literacy levels. Therefore, participants were divided into 2 groups for each study as follows:

- Study 1: (1) low eHealth literacy levels (≤27 points) and (2) high eHealth literacy levels (>32 points).
- Study 2: (1) low eHealth literacy levels (≤23 points) and (2) high eHealth literacy levels (>31 points).

In study 1 (n=80), 35 (43.8%) participants had low eHealth literacy levels, and 9 (11.3%) had high levels. In study 2 (n=301), 115 (38.2%) participants had low eHealth literacy levels, and 71 (23.6%) had high levels.

### Construct Validity: Known-Groups

We performed known-groups validity in study 2, as presented in the following subsections.

#### Association Between eHealth Literacy and Age (Student t test and Pearson Correlation)

To analyze the association between eHealth literacy and age, participants were distributed into two different groups: (1) 65-79 years and (2) ≥80 years. The first group comprised 269 (89.4%) participants, and the second group comprised 32 (10.6%) participants. The analysis showed differences statistically significant between eHealth literacy levels (total scale value) and age (t_299_=3.94; *P*<.001). Individuals aged 65-79 years had, on average, 7.0 points higher eHEALS scores than those aged ≥80 years. We also carried out a correlation analysis between both variables (Pearson correlation), which showed that variables are inversely related, which means the greater one variable, the smaller the other (*r*=–0.355, *P*<.001).

#### Association Between eHealth Literacy and Sex (Student t test)

Female respondents showed an average of 23.68 (SD 9.89) points and male respondents showed an average of 23.03 (SD 9.26) points; however, no statistically significant differences between eHealth literacy levels (total scale value) and sex were found (t_299_=0.59; *P*=.31).

#### Association Between eHealth Literacy and Residence Area (Student t test)

Statistically significant differences between eHealth literacy levels (total scale value) and residence area were found (t_299_=4.19; *P*<.001). Participants living in urban areas showed an average of 24.11 (SD 9.16) points, and those living in rural areas showed an average of 17.03 (SD 10.15) points.

#### Association Between eHealth Literacy and Education Level (1-Way ANOVA or Kruskal-Wallis Test)

Statistically significant differences between eHealth literacy levels (total scale value) and education level were found (*F*_8_=10.24; *P*<.001), with the highest mean values corresponding to the education levels of doctoral or equivalent level (ISCED 8; mean score of eHealth literacy levels: 31.57, SD 4.20), master’s degree or equivalent (ISCED 7; mean 29.17, SD 5.60), and postsecondary but not tertiary education (ISCED 4; mean 27.68, SD 8.52). Since some of the education levels had nonrepresentative samples (ie, ISCEDs 2, 4, 5, 7, and 8), we tested the association between eHealth literacy and education level using Kruskal-Wallis test (H=58.56; *P*<.001). In this analysis, we found statistically significant differences between participants with primary education (ISCED 1) and all other education levels, with an exception for lower secondary education (ISCED 2) and master’s degree or equivalent (ISCED 7). No other statistically significant differences were found.

The mean (SD) and significance of eHEALS-PT24 by subgroups (ie, age, sex, residence area, and education level) calculated in study 2 are presented in Table 8.

**Table 8:** Mean (SD) and significance of eHEALS-PT24^a^ score by subgroups (study 2; n=301).

Variable				Frequency, n (%)		eHEALS-PT24 score, mean (SD)		eHEALS-PT24, *P* value	
**Age group (years)**									<.001
		65-79		269 (89.37)		24.04 (9.23)			
		≥80		32 (10.63)		17.19 (9.99)			
**Sex**									.56
		Female		131 (43.52)		23.68 (9.89)			
		Male		170 (56.48)		23.03 (9.26)			
**Residence area**									<.001
		Urban		267 (88.7)		24.11 (9.16)			
		Rural		34 (11.3)		17.03 (10.15)			
**Education level**									<.001^c^
		ISCED^b^ 1: primary education		116 (38.54)		17.84 (9.91)			
		ISCED 2: lower secondary education		22 (7.31)		24.86 (8.67)			
		ISCED 3: upper secondary education		51 (16.94)		26.67 (7.25)			
		ISCED 4: postsecondary but not tertiary education		22 (7.31)		27.68 (8.52)			
	ISCED 5: short-cycle tertiary education		27 (8.97)		27.07 (6.71)				
	ISCED 6: bachelor’s degree or equivalent level		50 (16.61)		26.08 (7.62)				
	ISCED 7: master’s degree or equivalent level		6 (1.99)		29.17 (5.60)				
	ISCED 8: doctoral or equivalent level		7 (2.33)		31.57 (4.20)				

^a^eHEALS-PT24: Portuguese version of the eHealth Literacy Scale for older adults.

^b^ISCED: International Standard Classification of Education.

^c^ANOVA and Kruskal-Wallis.

## Discussion

### Principal Findings and Comparison With Previous Work

In this paper, we have presented the translation, cultural adaptation, and validation of the eHEALS for Portuguese older adults (eHEALS-PT24) by carrying out a general psychometric analysis in a sample of 80 participants (study 1) and a CFA including 301 participants (study 2). For this discussion, we highlight the findings obtained in study 2 since it represents the final stage of the validation of eHEALS-PT24, according to the followed protocol [49].

Based on scale and items stability and reliability analysis, we concluded that the eHEALS-PT24 is a reliable tool for measuring eHealth literacy. Correlations between all items of the eHEALS-PT24, ranging between moderate and strong ones, with statistical significance, indicated that the questions were related to the instrument with no redundancy. In addition, the instrument showed good internal consistency. All items were considered reliable to assess eHealth literacy levels in older adults, with high correlations between each other. Compared with the original validation study [10], internal consistency was higher (Cronbach α=0.88 vs 0.98), which is comparable with previous studies among older adults [24,36,39,43,44,48]. The only study showing a higher Cronbach α than ours was a validation in older Hispanic participants [48] (Cronbach α=0.99), but the sample was small (n=20), which was pointed out as a limitation by the authors.

Concerning construct validity, the eHEALS was originally developed with a single-factor structure [10], and in our study, the same structure was yielded in EFA, explaining 86.4% of the total variance. This total variance explained was higher compared with the original validation study [10] (56%), as well as to previous studies among older adults [24,36,39,43,44,48], suggesting the model captured a significant portion of the data’s variation. Regarding CFA, a single-factor model demonstrated high item loadings; however, the model fit was mixed: the chi-square test was significant (*P*<.001), CFI (0.94) and TLI (0.91) were both acceptable, and RMSEA (0.20) indicated a poor fit. The finding of a unidimensional structure also aligns with previous studies carried out in older adults [24,36,39]; however, it contradicts the results of the 2 other studies conducted among older adults, which indicated a better fit for the 3-factor structure [43,44]. The variability of factor structures in the literature and the RMSEA value obtained led us to compare the indices (ie, chi-square, CFI, TLI, and RMSEA) for 1-, 2-, and 3-factor structures. All indexes fitted well, but RMSEA remained poor (ie, 0.20 for 1 factor, 0.17 for 2 factors, and 0.18 for 3 factors), suggesting that the model may not fit the data well. These findings are in line with the eHEALS Korean validation study (CFI=0.95; TLI=0.92; and RMSEA=0.12) [36], which followed a unidimensional structure. Considering the original validation study [10], as well as others who used Rasch analysis (or other analyses under modern test theory) [25,40,41], we also decided on the 1-factor structure.

The eHEALS-PT24 mean score was 23.31, with 38.2% (115/301) of participants showing low eHealth literacy levels and 23.6% (71/301) showing high levels. Compared with other validation studies among older adults, the mean score was higher than in Spain (22.35) [48] but lower than in China (30.94) [39] and in the United States (29.05) [43]. As shown by previous studies [12,15,18,30,32], item 8 of the eHEALS-PT24 had the lowest average among all scale items, indicating that people do not feel so confident in using information from the internet to make health-related decisions compared with other eHealth literacy skills.

To further examine construct validity, known-groups validity was assessed, showing statistically significant differences in demographic variables (age, residence area, and education levels), except for sex. While a previous study among older adults [48] found no differences between eHealth literacy levels and age, we concluded that the greater the age, the smaller the eHealth literacy. Those aged 65-79 years had, on average, 7.0 points higher (eHEALS scores) than those aged ≥80 years. Regarding eHealth literacy levels and sex, Aponte and Nokes [48] pointed out significant differences, where mean values of eHEALS scores for men (13.85) were significantly lower than those in women (25.77). In our study, female respondents had, on average, 0.65 points higher eHEALS scores than male respondents; however, no statistically significant differences were found between eHealth literacy levels and sex (*P*=.31). We also tested the association of eHealth literacy levels and the residence area, finding statistically significant differences (*P*<.001), where participants living in urban areas had on average 7.08 points higher (eHEALS scores) than those living in rural areas. Regarding education levels, the highest mean values corresponded to doctoral or equivalent level (ISCED 8; 31.57), master’s degree or equivalent (ISCED 7; 29.17), and postsecondary but not tertiary education (ISCED 4; 27.68). Since some education-level cases had nonrepresentative samples (n<30), this result should be read with caution. Therefore, we can only infer participants with primary education (ISCED 1) had significant differences in eHealth literacy levels from the participants with other education levels, except for lower secondary education (ISCED 2) and master’s degree or equivalent (ISCED 7).

### Strengths and Limitations

There are some limitations in this study. First, there was limited geographic representation since the sample did not include older people from all regions of the country (only Faro and Lisbon). If we consider, however, the sample representativeness, we believe it is suitable for providing us with an adequate snapshot of our population of interest. Second, since the web-based questionnaires were only applied in the pilot study, we did not have any rigorous confirmatory process to determine that participants were aged, in fact, aged 65 years or older. Third, similarly to other validation studies [25,27,30-32,43,44,48], test-retest reliability was not conducted. As such, further research could address reliability and responsiveness over time. Fourth, although a more comprehensive psychometric analysis could be conducted to establish the instrument validity, such as investigating correlations with external measures of eHealth literacy or health outcomes, the results of this study are promising and show that the eHEALS-PT24 is a reliable tool for perceived measurement of eHealth literacy. Fifth, the eHEALS instrument measures a narrow scope of eHealth literacy and may not fully capture the complex concept of eHealth literacy nowadays since it was developed before the social media era (Web 2.0). Nevertheless, the benefits of eHEALS (strong psychometric properties, brevity, and usability) outweigh its shortcomings, and the eHEALS arguably serves as a convenient instrument for health professionals.

### Conclusions

In conclusion, the eHEALS-PT24 is a reliable and valid instrument for assessing and monitoring the eHealth literacy of Portuguese older adults. This instrument can be useful in identifying older adults who are able to use eHealth resources and participate in eHealth interventions aimed at engaging them in health care and helping them to manage their health and, by extension, assess the effects of eHealth interventions.

## Appendix

Multimedia Appendix 1 Portuguese version of the eHealth Literacy Scale for older adults (eHEALS-PT24).

## Abbreviations

CFA: confirmatory factor analysis
CFI: comparative fit index
EFA: exploratory factor analysis
eHEALS: eHealth Literacy Scale
eHEALS-PT24: Portuguese version of the eHealth Literacy Scale for older adults
ICT: information and communication technology
I-CVI: content validity index at the item level
ISCED: International Standard Classification of Education
KMO: Kaiser-Meyer-Olkin
RMSEA: root mean square error of approximation
S-CVI: content validity index at the scale level
TLI: Tucker-Lewis Index

## References

[1] (2014). European citizen's digital health literacy. European Commission.

[2] Portz JD (2017). A review of web-based chronic disease self-management for older adults. Gerontechnology.

[3] Buyl R, Beogo I, Fobelets M, Deletroz C, van Landuyt P, Dequanter S, Gorus E, Bourbonnais A, Giguère A, Lechasseur K, Gagnon M (2020). e-Health interventions for healthy aging: a systematic review. Syst Rev.

[4] Renzi E, Baccolini V, Migliara G, de Vito C, Gasperini G, Cianciulli A, Marzuillo C, Villari P, Massimi A (2022). The Impact of eHealth interventions on the improvement of self-care in Chronic patients: an overview of systematic reviews. Life (Basel).

[5] Stellefson M, Hanik B, Chaney B, Chaney D, Tennant B, Chavarria EA (2011). eHealth literacy among college students: a systematic review with implications for eHealth education. J Med Internet Res.

[6] Wilson J, Heinsch M, Betts D, Booth D, Kay-Lambkin F (2021). Barriers and facilitators to the use of e-health by older adults: a scoping review. BMC Public Health.

[7] Jung SO, Son YH, Choi E (2022). E-health literacy in older adults: an evolutionary concept analysis. BMC Med Inform Decis Mak.

[8] Health literacy: the solid facts. World Health Organization.

[9] Norman CD, Skinner HA (2006). eHealth literacy: essential skills for consumer health in a networked world. J Med Internet Res.

[10] Norman CD, Skinner HA (2006). eHEALS: the eHealth literacy scale. J Med Internet Res.

[11] Lee J, Lee E, Chae D (2021). eHealth literacy instruments: systematic review of measurement properties. J Med Internet Res.

[12] Mialhe FL, Moraes KL, Sampaio HADC, Brasil VV, Vila VDSC, Soares GH, Rebustini F (2021). Evaluating the psychometric properties of the eHealth literacy scale in brazilian adults. Rev Bras Enferm.

[13] Koo M, Norman C, Hsiao-Mei C (2012). Psychometric evaluation of a Chinese version of the eHealth literacy scale (eHEALS) in school age children. Int Electron J Health Educ.

[14] Chang A, Schulz PJ (2018). The measurements and an elaborated understanding of Chinese eHealth literacy (C-eHEALS) in chronic patients in China. Int J Environ Res Public Health.

[15] Ma Z, Wu M (2019). The psychometric properties of the Chinese eHealth literacy scale (C-eHEALS) in a Chinese rural population: cross-sectional validation study. J Med Internet Res.

[16] Xu RH, Zhou L, Lu SY, Wong EL, Chang J, Wang D (2020). Psychometric validation and cultural adaptation of the simplified Chinese eHealth literacy scale: cross-sectional study. J Med Internet Res.

[17] Shiferaw KB (2020). Validation of the ethiopian version of eHealth literacy scale (ET-eHEALS) in a population with chronic disease. Risk Manag Healthc Policy.

[18] Soellner R, Huber S, Reder M (2014). The concept of eHealth literacy and its measurement. J Media Psychol.

[19] Juvalta S, Kerry MJ, Jaks R, Baumann I, Dratva J (2020). Electronic health literacy in swiss-german parents: cross-sectional study of eHealth literacy scale unidimensionality. J Med Internet Res.

[20] Efthymiou A, Middleton N, Charalambous A, Papastavrou E (2019). Adapting the eHealth literacy scale for carers of people with chronic diseases (eHeals-Carer) in a sample of greek and cypriot carers of people with dementia: reliability and validation study. J Med Internet Res.

[21] Zrubka Z, Hajdu O, Rencz F, Baji P, Gulácsi L, Péntek M (2019). Psychometric properties of the hungarian version of the eHealth literacy scale. Eur J Health Econ.

[22] Wijaya MC, Kloping YP (2021). Validity and reliability testing of the indonesian version of the eHealth literacy scale during the COVID-19 pandemic. Health Informatics J.

[23] Bazm S, Mirzaei M, Fallahzadeh H, Bazm R (2016). Validity and reliability of Iranian version of eHealth literacy scale. J Community Health Res.

[24] Lin C, Broström A, Griffiths MD, Pakpour AH (2020). Psychometric evaluation of the persian eHealth literacy scale (eHEALS) among elder iranians with heart failure. Eval Health Prof.

[25] Diviani N, Dima AL, Schulz PJ (2017). A psychometric analysis of the Italian version of the eHealth literacy scale using item response and classical test theory methods. J Med Internet Res.

[26] del Giudice P, Bravo G, Poletto M, de Odorico A, Conte A, Brunelli L, Arnoldo L, Brusaferro S (2018). Correlation between eHealth literacy and health literacy using the eHealth literacy scale and real-life experiences in the health sector as a proxy measure of functional health literacy: cross-sectional web-based survey. J Med Internet Res.

[27] van der Vaart R, van Deursen AJ, Drossaert CH, Taal E, van Dijk JA, van de Laar MA (2011). Does the eHealth Literacy Scale (eHEALS) measure what it intends to measure? Validation of a dutch version of the eHEALS in two adult populations. J Med Internet Res.

[28] Brørs G, Wentzel-Larsen T, Dalen H, Hansen TB, Norman CD, Wahl A, Norekvål TM (2020). Psychometric properties of the norwegian version of the electronic health literacy scale (eHEALS) among patients after percutaneous coronary intervention: cross-sectional validation study. J Med Internet Res.

[29] Dale JG, Lüthi A, Fundingsland Skaraas B, Rundereim T, Dale B (2020). Testing measurement properties of the norwegian version of electronic health literacy scale (eHEALS) in a group of day surgery patients. J Multidiscip Healthc.

[30] Duplaga M, Sobecka K, Wójcik S (2019). The reliability and validity of the telephone-based and online polish eHealth literacy scale based on two nationally representative samples. Int J Environ Res Public Health.

[31] Burzyńska J, Rękas M, Januszewicz P (2022). Evaluating the psychometric properties of the eHealth literacy scale (eHEALS) among polish social media users. Int J Environ Res Public Health.

[32] Tomás C, Queirós P, Ferreira T (2014). Analysis of the psychometric properties of the portuguese version of an eHealth literacy assessment tool. Rev Enf Ref (IV serie).

[33] Gazibara T, Cakic J, Cakic M, Pekmezovic T, Grgurevic A (2019). eHealth and adolescents in serbia: psychometric properties of eHeals questionnaire and contributing factors to better online health literacy. Health Promot Int.

[34] Chung S, Park BK, Nahm E (2018). The korean eHealth literacy scale (K-eHEALS): reliability and validity testing in younger adults recruited online. J Med Internet Res.

[35] Gartrell K, Han K, Trinkoff A, Cho H (2020). Three-factor structure of the eHealth literacy scale and its relationship with nurses' health-promoting behaviours and performance quality. J Adv Nurs.

[36] Kim H, Yang E, Ryu H, Kim HJ, Jang SJ, Chang SJ (2021). Psychometric comparisons of measures of eHealth literacy using a sample of Korean older adults. Int J Older People Nurs.

[37] Pérez G, Almagro BJ, Hernando Gómez Á, Aguaded Gómez JI (2015). Validácion de la escala eHealth literacy (eHEALS) en población universitaria española. Rev Esp Salud Publica.

[38] Wångdahl J, Jaensson M, Dahlberg K, Nilsson U (2020). The swedish version of the electronic health literacy scale: prospective psychometric evaluation study including thresholds levels. JMIR Mhealth Uhealth.

[39] Chung S, Nahm E (2015). Testing reliability and validity of the eHealth literacy scale (eHEALS) for older adults recruited online. Comput Inform Nurs.

[40] Nguyen J, Moorhouse M, Curbow B, Christie J, Walsh-Childers K, Islam S (2016). Construct validity of the eHealth literacy scale (eHEALS) among two adult populations: a rasch analysis. JMIR Public Health Surveill.

[41] Paige SR, Krieger JL, Stellefson M, Alber JM (2017). eHealth literacy in chronic disease patients: An item response theory analysis of the eHealth literacy scale (eHEALS). Patient Educ Couns.

[42] Richtering SS, Morris R, Soh S, Barker A, Bampi F, Neubeck L, Coorey G, Mulley J, Chalmers J, Usherwood T, Peiris D, Chow CK, Redfern J (2017). Examination of an eHealth literacy scale and a health literacy scale in a population with moderate to high cardiovascular risk: Rasch analyses. PLoS One.

[43] Stellefson M, Paige SR, Tennant B, Alber JM, Chaney BH, Chaney D, Grossman S (2017). Reliability and validity of the telephone-based eHealth literacy scale among older adults: cross-sectional survey. J Med Internet Res.

[44] Sudbury-Riley L, FitzPatrick M, Schulz PJ (2017). Exploring the measurement properties of the eHealth literacy scale (eHEALS) among baby boomers: a multinational test of measurement invariance. J Med Internet Res.

[45] Paige SR, Miller MD, Krieger JL, Stellefson M, Cheong J (2018). Electronic health literacy across the lifespan: measurement invariance study. J Med Internet Res.

[46] Hyde LL, Boyes AW, Evans T, Mackenzie LJ, Sanson-Fisher R (2018). Three-factor structure of the eHealth literacy scale among magnetic resonance imaging and computed tomography outpatients: a confirmatory factor analysis. JMIR Hum Factors.

[47] Holch P, Marwood JR (2020). EHealth literacy in UK teenagers and young adults: exploration of predictors and factor structure of the eHealth literacy scale (eHEALS). JMIR Form Res.

[48] Aponte J, Nokes KM (2017). Electronic health literacy of older Hispanics with diabetes. Health Promot Int.

[49] Sousa VD, Rojjanasrirat E (2011). Translation, adaptation and validation of instruments or scales for use in cross-cultural health care research: a clear and user-friendly guideline. J Eval Clin Pract.

[50] Clark-Carter D (2010). Quantitative Psychological Research. The Complete Student's Companion. 3rd edition.

[51] Hu L, Bentler PM (1999). Cutoff criteria for fit indexes in covariance structure analysis: conventional criteria versus new alternatives. Structural Equation Modeling: A Multidisciplinary Journal.

