# Supplementary material for: Psychometric Analysis of the eHealth Literacy Scale in Portuguese Older Adults (eHEALS-PT24): Instrument Development and Validation
Source: J Med Internet Res. 2025 Feb 26;27:e57730. doi: 10.2196/57730 (PMC11904376; doi:10.2196/57730)
Supplement: Multimedia Appendix 1 [file jmir_v27i1e57730_app1.pdf]

Multimedia Appendix 1
Portuguese version of the eHealth Literacy Scale for older adults (eHEALS-PT24).

Item	**Portuguese translation**[a]	Original version[b]
PT24 – eHEALS1	Eu sei quais os recursos de saúde disponíveis na internet.	I know what health resources are available on the Internet.
PT24 – eHEALS2	Eu sei onde encontrar recursos de saúde úteis na Internet.	I know where to find helpful health resources on the Internet.
PT24 – eHEALS3	Eu sei como encontrar recursos de saúde úteis na Internet.	I know how to find helpful health resources on the Internet.
PT24 – eHEALS4	Eu sei como utilizar a Internet para responder às minhas questões sobre saúde.	I know how to use the Internet to answer my questions about health.
PT24 – eHEALS5	Eu sei como utilizar as informações de saúde que encontro na Internet para me ajudar.	I know how to use the health information I find on the Internet to help me.
PT24 – eHEALS6	Eu tenho as habilidades necessárias para avaliar os recursos de saúde que encontro na Internet.	I have the skills I need to evaluate the health resources I find on the Internet.
PT24 – eHEALS7	Eu consigo distinguir recursos de saúde de alta qualidade de recursos de saúde de baixa qualidade na Internet.	I can tell high quality health resources from low quality health resources on the Internet.
PT24 – eHEALS8	Eu sinto-me confiante em utilizar informações da Internet para tomar decisões de saúde.	I feel confident in using information from the Internet to make health decisions.

[a]As in the original version (English), answers are on a 5-point Likert scale ranging from 1 “discordo totalmente” (“strongly disagree”) to 5 “concordo totalmente” (“strongly agree”); [b]Source: Norman, C. D., & Skinner, H. A. (2006a). eHEALS: The eHealth literacy scale. Journal of Medical Internet Research, 8(4), e27.
